# Biomimetic multi-resolution analysis for robust speaker recognition

**DOI:** 10.1186/1687-4722-2012-22

**Published:** 2012-09-07

**Authors:** Sridhar Krishna Nemala, Dmitry N Zotkin, Ramani Duraiswami, Mounya Elhilali

**Affiliations:** 1 Department of Electrical and Computer Engineering, Center for Language and Speech Processing, Johns Hopkins University, Baltimore, MD, USA; 2 Institute for Advanced Computer Studies, University of Maryland, College Park, MD, USA

## Abstract

Humans exhibit a remarkable ability to reliably classify sound sources in the environment even in presence of high levels of noise. In contrast, most engineering systems suffer a drastic drop in performance when speech signals are corrupted with channel or background distortions. Our brains are equipped with elaborate machinery for speech analysis and feature extraction, which hold great lessons for improving the performance of automatic speech processing systems under adverse conditions. The work presented here explores a biologically-motivated multi-resolution speaker information representation obtained by performing an intricate yet computationally-efficient analysis of the information-rich spectro-temporal attributes of the speech signal. We evaluate the proposed features in a speaker verification task performed on NIST SRE 2010 data. The biomimetic approach yields significant robustness in presence of non-stationary noise and reverberation, offering a new framework for deriving reliable features for speaker recognition and speech processing.

## Introduction

In addition to the intended message, human voice carries the unique imprint of a speaker. Just like finger-prints and faces, voice prints are biometric markers with tremendous potential for forensic, military, and commercial applications [1]. However, despite enormous advances in computing technology over the last few decades, automatic speaker verification (ASV) systems still rely heavily on training data collected in controlled environments, and most systems face a rapid degradation in performance when operating under previously unseen conditions (e.g. channel mismatch, environmental noise, or reverberation). In contrast, human perception of speech and ability to identify sound sources (including voices) is quite remarkable even at relatively high distortion levels [2]. Consequently, the pursuit of human-like recognition capabilities has spurred great interest in understanding how humans perceive and process speech signals.

One of the intriguing processes taking place in the central auditory system involves ensembles of neurons with variable tuning to spectral profiles of acoustic signals. In addition to the frequency (tonotopic) organization emerging as early as the cochlea, neurons in the central auditory system (specifically in the midbrain and more prominently in the auditory cortex) exhibit tuning to a variety of filter bandwidths and shapes [3]. This elegant neural architecture provides a detailed multi-resolution analysis of the spectral sound profile, which is presumably relevant to speech and speaker recognition. Only few studies so far have attempted to use this cortical representation in speech processing, yielding some improvements for automatic speech recognition at the expense of *substantial computational complexity* [4,5]. To the best of our knowledge, no similar work was done in ASV.

In the present report, we explore the use of a multi-resolution analysis for robust speaker verification. Our representation is simple, effective, and computationally-efficient. The proposed scheme is carefully optimized to be particularly sensitive to the information-rich spectro-temporal attributes of the signal while maintaining robustness to unseen noise distortions. The choice of model parameters builds on our current knowledge of psychophysical principles of speech perception in noise [6,7] complemented with a statistical analysis of the dependencies between spectral details of the message and speaker information. We evaluate the proposed features in an ASV system and compare it against one of the best performing systems in NIST 2010 SRE evaluation [8] under detrimental conditions such as white noise, non-stationary additive noise, and reverberation.

The following section describes details of the proposed multi-resolution spectro-temporal model. It is followed by an analysis that motivates the choice of model parameters to maximize speaker information retention. Next, we describe the experimental setup and results. We finish with a discussion of these results and comment on potential extensions towards achieving further noise robustness.

## The biomimetic multi-resolution analysis

**Figure 1:**
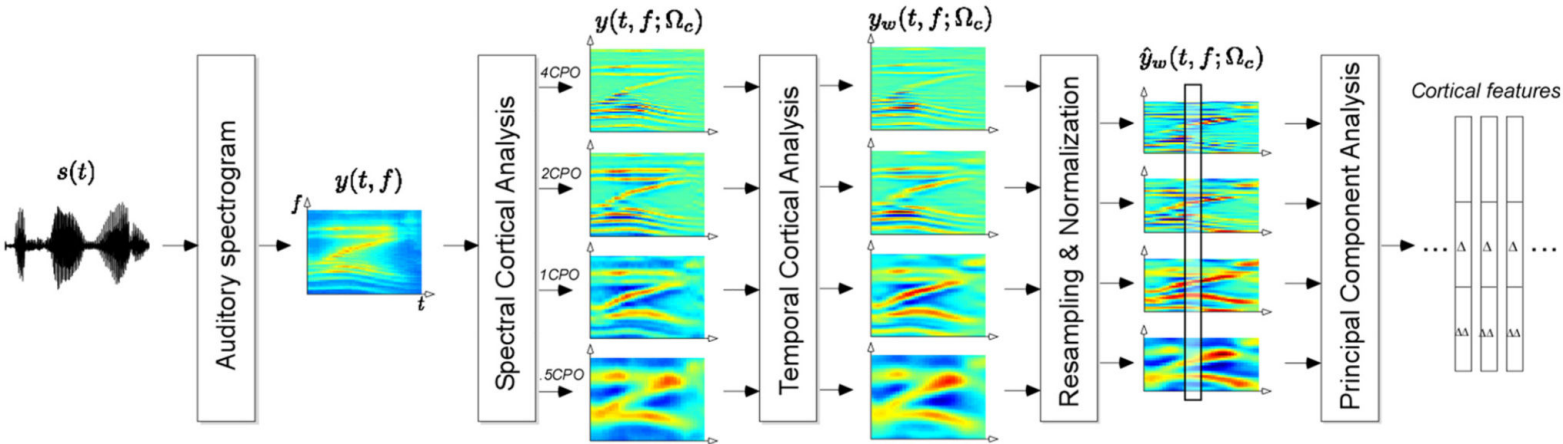
An outline of the cortical features extraction algorithm A schematic diagram of the algorithm that transforms a speech waveform into a sequence of cortical feature vectors.

An overview of the processing chain described in this section is presented in Figure 1.

### Peripheral analysis

The speech signal is processed through a pre-emphasis stage (implemented as a first-order high pass filter with pre-emphasis coefficient 0.97), and a time-frequency auditory spectrogram is generated using a biomimetic sound processing model described in details in [9] and briefly summarized here (Equation 1). First, the signal *s*(*t*) undergoes a cochlear frequency analysis modeled by a bank of 128 constant-*Q* (*Q* = 4) highly asymmetric band-pass filters *h*(*t*; *f*) equally spaced over the span of 5^1^/_3_ octaves on a logarithmic frequency axis. The filterbank output is a spatiotemporal pattern of cochlea basilar membrane displacements *y*_coch_(*t*, *f*) over 128 channels. Next, a lateral inhibitory network detects discontinuities in the responses across the tonotopic (frequency) axis, resulting in further filterbank frequency selectivity enhancement. This step is modeled as a first-order differentiation operation across the channel array followed by a half-wave rectifier and a short-term integrator. The temporal integration window is given by *μ*(*t*; *τ*) = *e*^−*t/τ*^ *u*(*t*) with time constant *τ* = 10 ms mimicking the further loss of phase-locking observed in the midbrain. This time constant controls the frame rate of the spectral vectors. Finally, a nonlinear cubic root compression of the spectrum is performed, resulting in an auditory spectrogram *y*(*t*, *f*):

$$y_{\text{coch}}(t,f)=s(t){⊗}_{t}h(t;f),$$

(1) $$y_{\text{lin}}(t,f)=\text{max}(\partial_{t}y_{\text{coch}}(t,f),0),$$

$$y(t,f)={\left[y_{\text{lin}}(t,f){⊗}_{t}\mu (t;\tau )\right]}^{1/3},$$

where ⊗_*t*_ represents convolution with respect to time. The choice of the auditory spectrogram is motivated by its neurophysiological foundation as well as its proven self-normalization and robustness properties (see [10] for full details).

### Spectral cortical analysis

**Figure 2:**
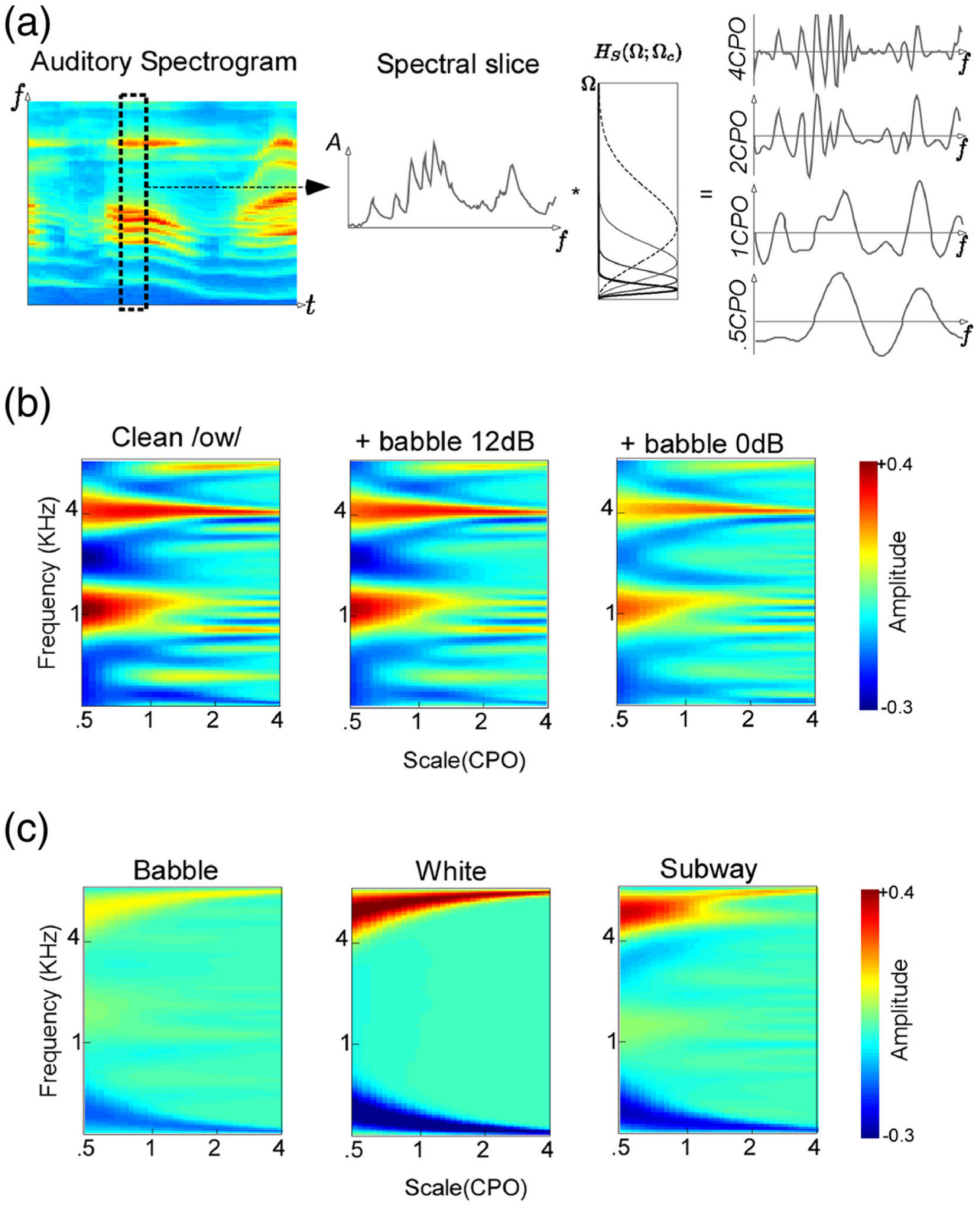
Details of the speech spectral analysis **(a)** The speech spectrogram is analyzed separately at each time instant. Each spectrogram slice is filtered through a bandpass filter *H_S_*(Ω; Ω_*c*_) parameterized by Ω_*c*_. The * operator signifies the filtering operation. Four such filtering operations yield four views of the same spectral slice; each view highlights different details about the spectrum, notably formant peaks and harmonic structure. **(b)** Cortical features for clean and noisy versions of one phoneme \ow\. The plots show magnitude as a function of frequency and scale. For visualization, the discrete image points have been interpolated in MATLAB using a bicubic interpolation routine. Notice the consistency of formant peaks around 1 and 4 KHz and of harmonic energies at 2 CPO and 4 CPO despite the additive noise distortion. **(c)** Cortical features for different types of additive noise. Note that the patterns exhibited are quite different. Subtle peaks due to harmonicity and formant structure of human speech can be seen in the left panel (babble noise).

The auditory spectrogram is processed further in order to capture the spectral details present in each spectral slice. The processing is based on neurophysiological findings that neurons in the central auditory pathway are tuned not only to frequencies but also to spectral shapes, in particular to peaks of various widths on the log-frequency axis [3,11,12]. The spectral width is characterized by a parameter called scale and is measured in cycles per octave, or CPO. Physiological data indicates that auditory cortex neurons are highly scale-selective, thus expanding the cochlear one-dimensional tonotopic axis onto a two-dimensional sheet that explicitly encodes tonotopy as well as spectral shape details (see Figures 1 and 2).

The cortical analysis is implemented using a bank of modulation filters operating in the Fourier domain. The algorithm processes each data frame individually. The Fourier transform of each spectral slice *y*(*t*_0_, *f*) is multiplied by a modulation filter *H_S_*(Ω; Ω_c_) that is tuned to spectral features of scale Ω_*c*_. The filtering operates on the magnitude of the signal. After filtering, the inverse Fourier transform is performed and the real part is taken as the new filtered slice. This process is then repeated with a number of different Ω_*c*_, yielding a number of filtered spectrograms *y*(*t*, *f*; Ω_*c*_), each with features of scale Ω_*c*_ emphasized (see Figure 1). This set of spectrograms constitutes the spectral cortical representation of the sound.

The filter *H_S_* (Ω; Ω_*c*_) is defined as

(2) $$H_{S}(\Omega ;\Omega_{c})={\left(\Omega /\Omega_{c}\right)}^{2}e^{\left[1-{\left(\Omega /\Omega_{c}\right)}^{2}\right]},\;0\leq \Omega \leq \Omega_{\text{max}},$$

where Ω_max_ is the highest spectral modulation frequency (set at 12 CPO given our spectrogram resolution of 24 channels per octave).

### Choice of spectral parameters

**Figure 3:**
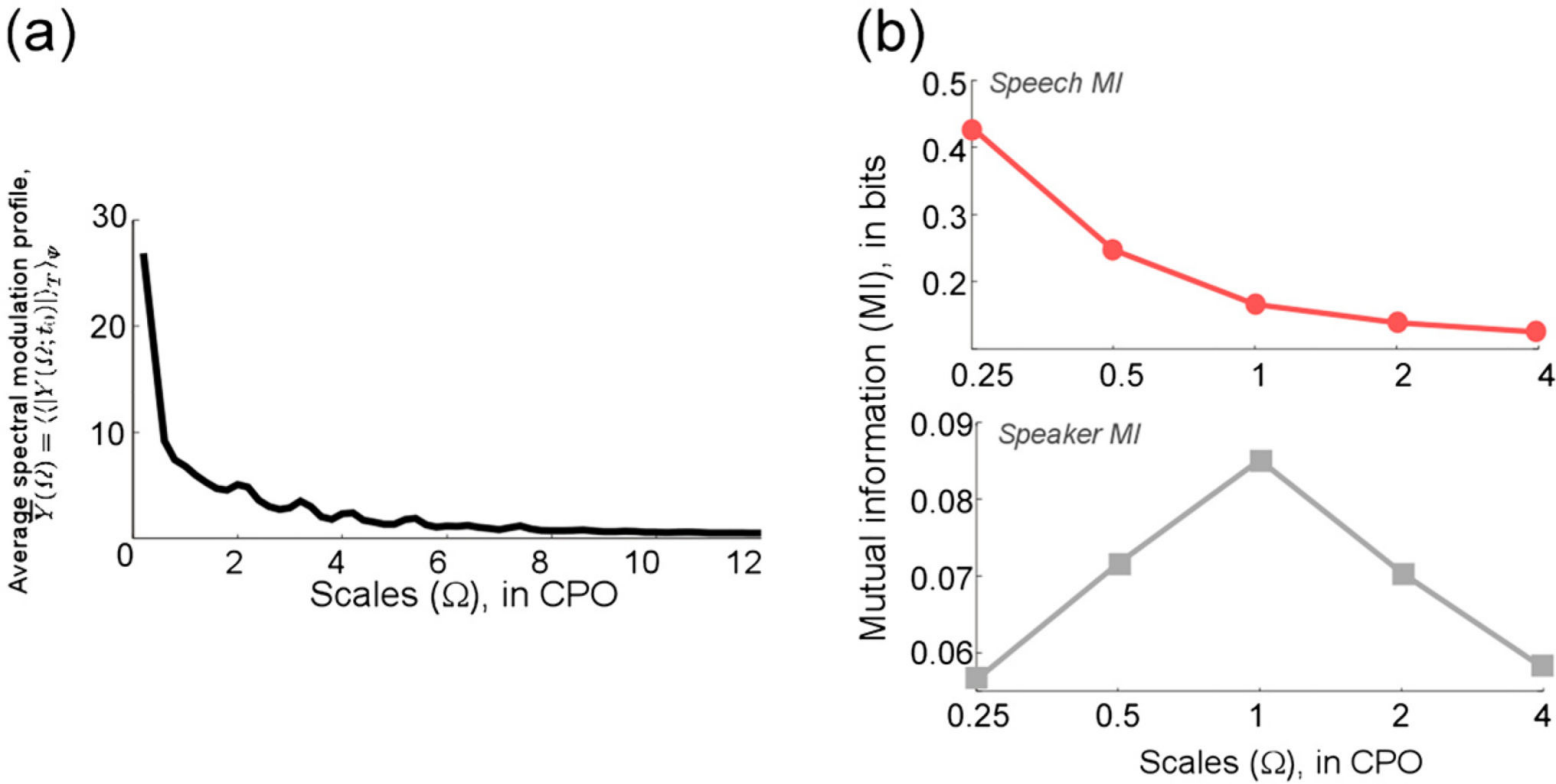
Speech signal spectral analysis **(a)** Average spectral modulation profile *Ȳ*(Ω) = 〈〈|*Y*(Ω; *t*_0_)|〉_*T*_〉_Ψ_; **(b)** Top panel: MI between feature representation and speech message as a function of scale. Bottom panel: MI between feature representation and speaker identity as a function of scale.

The set of scales Ω_*c*_ is chosen by dividing the spectral modulation axis into equal energy regions using a training corpus (TIMIT database [13]) as described below. Define the average spectral modulation profile *Ȳ*(Ω) = 〈〈|*Y*(Ω; *t*_0_)|〉_*T*_〉_Ψ_ as the ensemble mean of the magnitude Fourier transform of the spectral slice *y*(*t*_0_, *f*) averaged over all times *T* and over entire speech corpus Ψ. The resulting ensemble profile (shown in Figure 3a) is then divided into *M* equal energy regions Γ_*k*_:

(3) $$\Gamma_{k}=\int_{\Omega_{k}}^{\Omega_{k+1}}\bar{Y}(\Omega )d\Omega ,\;\Gamma_{k}=\Gamma_{k+1},\;k=1,\ldots ,M-1,$$

where Ω_*k*_ and Ω_*k*+1_ denote the lower and upper cutoffs for *k*th band, Ω_1_ = 0, and Ω_*M*_ = 4.^a^ This sampling scheme ensures that the high energy regions are sampled more densely, which has the dual advantage of sampling the given modulation space with a relatively small set of scales and emphasizing high-energy signal components, which are presumably noise-robust. Setting *M* = 5 results in cutoffs at {0.18, 0.59, 1.34, 2.36, 4}, which are approximated to the nearest log-scale as Ω_*c*_ = {0.25, 0.5, 1.0, 2.0, 4.0}. Finally, in order to put less emphasis on message-dominant regions of the spectrum, we drop the 0.25 CPO filter, which carries mostly articulatory and formant-specific information relevant to the speech message (analysis presented in the next section). The remaining set of Ω_*c*_ = {0.5, 1.0, 2.0, 4.0} is found to be a good tradeoff between computational complexity and system performance.

### Temporal filtration

In this stage, the spectral cortical features are processed through a bandpass temporal modulation filter to remove information that is believed to be mostly irrelevant. It was shown in [14] that the neurons in the auditory cortex are mostly sensitive to the modulation rates between 0.5 and 12 Hz and that the same modulation range represents the information crucial for speech comprehension [7]. Accordingly, the filtering is performed by multiplying the Fourier transform of the time sequence of each spectral feature by a bandpass filter *H_T_*(*w*; *w_l_*, *w_h_)*:

$$H_{T}(w;w_{l},w_{h}])={\left(\alpha w\right)}^{2}e^{\left[1-{\left(\alpha w\right)}^{2}\right]},$$

(4) $$\alpha =\{\begin{array}{cc} 1/w_{l}, & 0\leq w<w_{l},\\ 1/w, & w_{l}\leq w\leq w_{h},\\ 1/w_{h}, & w_{h}<w\leq w_{\text{max}}, \end{array}$$

where *w_l_* = 0.5Hz, *w_h_* = 12.0Hz, *w*_max_ = 1/(2*t_f_*), and *t_f_* = 10 ms (the frame length). After filtering in Fourier domain, the inverse Fourier transform is performed and the real part of the output forms the temporally filtered spectral cortical representation of the sound *y_w_*(*t*, *f*; Ω_*c*_). This operation is performed on an utterance by utterance basis.

### Cortical features

To reduce computational complexity and to allow use of state-of-the-art speaker verification machinery (which generally expects a relatively low-dimensional input), the spectral cortical representation is downsampled in frequency by a factor of 4 (Figure 1). The resulting feature representation has a dimensionality of 128 (32 auditory frequency channels multiplied by four scales used for analysis). The features are then normalized to zero mean and unit variance for each utterance, yielding the reduced set of spectrograms *ŷ_w_*(*t*, *f*; Ω_*c*_). Principal component analysis is used to further reduce the feature dimensionality to 19. This number is chosen for consistency with the dimensionality of the standard Mel-Frequency Cepstral Coefficients (MFCC) feature set used for speaker recognition. The reduced features, along with their first- and second-order derivatives, form the final 57-dimensional cortical feature vector used for the speaker verification task.

## Speech information versus speaker information

The speech signal carries both speech message and speaker identity information in distinct yet overlapping components. Separation of these elements is a non-trivial task in general. In the multi-resolution framework presented above, the broadest filters (0.25 and 0.5 CPO) capture primarily the overall spectral profile and formant peaks, while the others (1, 2, and 4 CPO) reflect narrower spectral details such as harmonics and subharmonic structure. In order to select a set of scales (Ω_*c*_) that are most relevant for the speaker recognition task, we analyze the mutual information (MI) between the feature vector (*X*), the speech message (*Y*_1_), and the speaker identity (*Y*_2_). The MI is a measure of the statistical dependence between random variables [15] and is defined for two discrete random variables *X* and *Y* as

(5) $$I(X;Y_{i})=\sum_{x\in X,y\in Y_{i}}p(x,y){\text{ log}}_{2}\frac{p(x,y)}{p(x)p(y)}.$$

To estimate the MI, the continuous feature vector is quantized by dividing its support into cells of equal volume. To characterize the speech message, phoneme labels from the TIMIT corpus are first divided into four broad phoneme classes. The variable *Y*_1_ thus takes four discrete values representing the phoneme categories: vowels, stops, fricatives, and nasals. The average MI (taken as the mean MI across all the frequency bands for a given scale) between the feature vector and the speech message is shown in Figure 3b (top) as a function of scale. For the speaker identity MI test, the TIMIT “sa1” speech utterance (*She had your dark suit in greasy wash water all year*) spoken by 100 different subjects is used; thus, *Y*_2_ takes 100 discrete values representing the speaker. The average MI between the feature vector and the speaker identity is shown in Figure 3b (bottom), again as a function of scale.^b^

Notice that while the lower scale (0.25 CPO) clearly provides significantly more information about the underlying linguistic message, the MI peak in Figure 3c (bottom) is centered at 1 CPO, highlighting the significance of pitch and harmonically-related frequency channels in representing speaker-specific information. In order to put less emphasis on message-carrying features of the speech signal, we drop the 0.25 CPO filter at the feature encoding stage for our ASV system and choose Ω_*c*_ = {0.5, 1.0, 2.0, 4.0} CPO.^c^

## Experiments and results

### Recognition setup

Text independent speaker verification experiments are conducted on the NIST 2010 speaker recognition evaluation (SRE) data set [8]. The *extended core* task of the evaluation involves 6.9 million trials broken down into nine common conditions reflecting a variety of channel mismatch scenarios [8] (see Table 1).

The front end of the implemented ASV system uses either the 57-dimensional MFCC feature vector or the 57-dimensional cortical feature vector. The MFCC feature vector is computed by invoking RASTAMAT “melfcc” function with ‘numcep’ parameter set to 20, dropping the first (energy) component of the output, and appending first- and second-order derivatives of the resultant feature vector. The cortical feature vector is obtained as described in the previous sections. For fair comparison between MFCC and cortical features, MFCC was supplemented with mean subtraction, variance normalization, and RASTA filtering [16] applied at the utterance level. Such processing parallels the temporal filtering and normalization performed on cortical features. A combination of ASR output provided by NIST and an in-house energy-based VAD system is used to drop all non-speech frames from input data.

The back-end is a robust state-of-the-art UBM-GMM system [17,18]. In a UBM-GMM system, each speaker’s distribution of feature vectors is modeled as a mixture of Gaussians, forming a Gaussian mixture speaker model (GMSM). In addition, a universal background model (UBM) defines a “generic” speaker. The UBM typically has hundreds of thousands of parameters and is trained on a very large amount of data (hundreds of hours of speech), which should include speech produced by a large number of individual speakers (in our case, the 2048-center diagonal-covariance UBM is trained on NIST SRE 2004, 2005, 2006, and 2008; Fisher; Switchboard-2; and Switchboard-Cellular databases).As the amount of speech available per individual speaker is typically much less than required to train the speaker model from scratch, the GMSM is produced by adapting UBM means so that the resulting model best describes the available speaker data. Finally, given the UBM, the candidate GMSM, and the audio file, the system extracts the feature vectors from the audio file and computes the log-likelihoods of these feature vectors belonging to the GMSM and to the UBM. The difference between these log-likelihoods constitutes the output score for this particular trial.

Our ASV system additionally employs the technique known as joint factor analysis [19,20]. JFA use enables channel variability compensation by offsetting the channel effects and more robust speaker model estimation by using more informative prior on speaker model distribution. To use JFA in the described framework, an alternative representation of the speaker model—a single vector *Z* (“supervector”)—is formed by concatenaging all GMSM means. JFA is trained in advance on a large annotated collection of audio files to learn the channel subspace (the basis over which *Z* preferentially varies when the same speaker’s voice is presented over different channels) and the speaker subspace (the basis over which *Z* preferentially varies when different speakers are presented over the same channel). In our system, the dimensionalities of speaker subspace and of channel subspace are 300 and 150, respectively. Then, when processing the previously unseen data, components of inter-speaker differences attributable to speaker/to channel are emphasized/canceled, respectively. This is done by projecting corresponding supervectors into speaker/channel subspaces, using speaker subspace projection of *Z* to modify GMSM, using channel subspace projection of *Z* to modify UBM, and performing scoring with these modified GMSM and UBM. Also, as the log-likelihood calculation is expensive, in our system an approximation to it is computed based on an inner product [20] is used.

Finally, the obtained scores are subject to ZT-normalization [21], and the decision threshold minimizing equal error rate (EER) is chosen (separately for each condition).

### Noise conditions

Every trial in NIST SRE 2010 consists of computing the matching score between a speaker model and an audio file. To evaluate the noise robustness of the proposed cortical features, several distorted versions of these audio files are created by adding different types of noise reflecting a variety of real world scenarios:

- White noise at signal-to-noise ratio (SNR) levels from 24 to 0 dB in 6 dB steps;
- Babble noise (from Aurora database [22]), same SNR levels;
- Subway noise (from Aurora database [22]), same SNR levels;
- Simulated reverberation with *RT*_60_ from 200 to 1,200 ms in steps of 200 ms.

It is important to mention that all training (UBM, JFA, and speaker model training) is done exclusively on clean data, and only the test audio files are corrupted. Note also that the train-test mismatch created by addition of noise/reverberation is superimposed on the train-test mismatch inherent to the SRE 2010 data.

### Results

**Figure 4:**
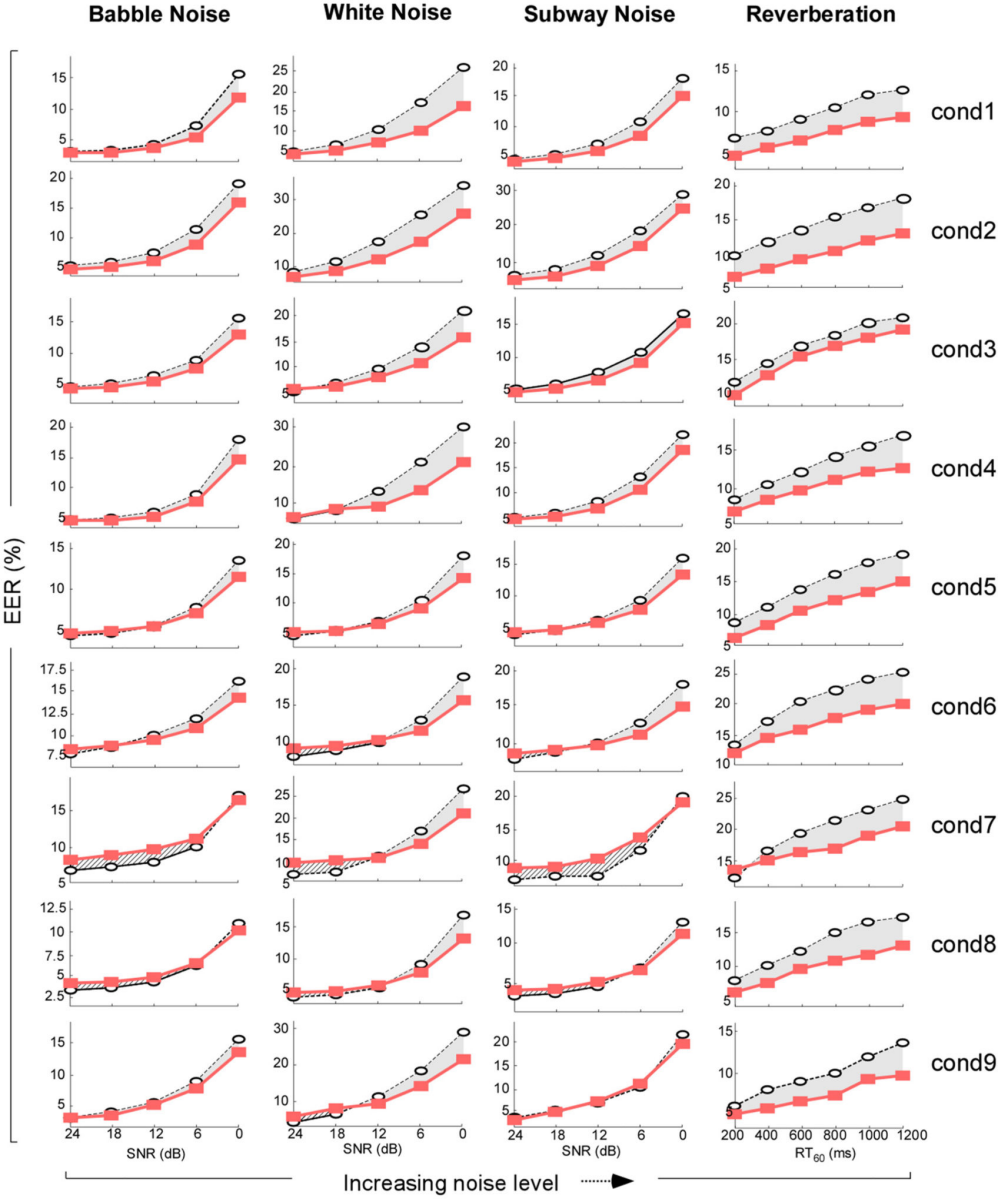
Evaluation results Performance of the proposed cortical features (red filled squares) and enhanced MFCC features (black open circles) on NIST SRE 2010 “extended core” database as a function of noise level, noise type, and condition. In each subplot, the noise level is shown on *X* axis and the EER (in percents) is on *Y* axis. Columns and rows of subplots belong to the same noise type and to the same condition, respectively. Note the *Y*-axis ranges are not the same in the subplots.

Figure 4 shows the speaker verification performance in terms of EER for the cortical features and for the MFCC features as a function of noise type/strength and trial condition. The results clearly demonstrate that the proposed cortical features provide substantially lower EER than the MFCC as noise level increases, indicating their robustness. The average performance for each noise type and trial condition is shown in Table 2. On average (across all conditions and all noise types), the cortical-features-based system yields 15.9% relative EER improvement over the robust state-of-the-art MFCC system. It is worth noting that the proposed approach is outperformed by the MFCC-based approach in only 4 out of the 36 cases. Because the proposed metric incorporates both a biomimetic auditory spectrogram previously shown to exhibit some noise-robustness characteristics [10] as well as multiresolution decomposition, we investigated further the contribution of both components in the reported improvements. We tested the system using the auditory spectrogram alone or an adaptation of the auditory spectrogram described here, coupled with a cepstral transformation. Neither system performed as well as the proposed multiresolution decomposition, hence strengthening the claim that our proposed multiresolution analysis is indeed responsible for the performance improvements shown in Table 2.

In some ASV applications, metrics other than EER may be more relevant. For example, in certain biometric speaker verification systems the key requirement is a low false alarm rate. We present our results here in terms of two additional metrics more suitable in such case, namely Miss-10 and quadratic DCF (decision cost function) metrics. These two metrics were used in the NIST 2011 IARPA BEST program SRE [23]. The Miss-10 metric is defined as the false alarm rate *P*_FA_ obtained when the decision threshold is set such that the miss rate *P*_Miss_ = 10%, and the quadratic DCF is defined as

(6) $$\text{DCF}=C_{\text{Miss}}\times {P_{\text{Miss}}}^{2}\times P_{\text{target}}+C_{\text{FA}}\times P_{\text{FA}}\times (1-P_{\text{targrt}})$$

with the parameter values *C*_Miss_ = 100, *C*_FA_ = 10, and *P*_target_ = 0.01.

The average verification performance for each noise type using the Miss-10 and quadratic DCF metrics is shown in Tables 3 and 4, respectively. As seen from the data, in the low false alarm region the proposed cortical features outperform the robust state-of-the-art MFCC system with even larger margin: 28.8% relative using the Miss-10 metric and 22.6% relative using the quadratic DCF metric.

## Discussion and conclusions

In this report, we explore the applicability of a multi-resolution analysis of speech signals to ASV. This framework maps the speech signal onto a rich feature space, highlighting and separating information about the glottal excitation signal, glottal shape, vocal tract geometry, and articulatory configuration (as each of these elements is an underlying factor for features of different width located in different areas on the log-frequency axis; see e.g. [24]). The cortical representation can be viewed as a “local” variant (w.r.t. log-frequency axis) of the analysis provided by MFCC analysis. This analogy stems from the fact that MFCC roughly correspond to spectral features of different widths integrated over the whole frequency range. In this work, both the “global-integration” MFCC approach and the “local” cortical approach are tested in a state-of-the-art ASV system on the NIST SRE 2010 dataset. While both perform comparably in clean condition, the cortical features are substantially more robust on noisy data, including non-stationary distortions as well as reverberation.

One of the intuitions behind the robustness observed in the proposed features is the fact that speech and noise generally exhibit different spectral shapes while occupying an overlapping spectral range. The expansion of the spectral axis with the multi-resolution analysis allows the extrication of some speech components from the masking noise, suppressing the noise components and providing for increased robustness. Furthermore, by highlighting the range between 0.5 and 4 CPO, the model stresses the most speaker-informative regions in the speech spectrum, which in turn map onto a modulation space to which humans are highly sensitive [7]. Such range is also commensurate with neurophysiological tuning observed in mammalian auditory cortex with most neurons concentrated around a spectral tuning of the order of few CPOs [3,14]. A similar emphasis is put on the temporal dynamics of the signal by underscoring the region between 0.5 and 12Hz, which defines natural boundaries for speech perception in noise by human listeners [7,25–28] and mostly coincides with temporal tuning of mammalian cortical neurons [14]. Higher temporal modulation frequencies represent mostly the syllabic and segmental rate of speech [2].

Unlike comparable multi-resolution schemes recently developed [4,5], the proposed approach does not involve dimension-expanded representations (close to 30,000 dimensions, which inherently require computationally-expensive schemes and therefore have limited applicability). Instead, our model is constrained to lie in a perceptually-relevant spectral modulation space and further uses a careful sampling scheme to encode the information with only four spectral analysis filters. This has the dual advantage of producing a feature space that is both low-dimensional and highly robust. The careful optimization of model parameters is necessary to strike a balance between simple and efficient computation and noise robustness.

Importantly, in our approach no model components have been customized in any way to deal with a specific noise condition, making it suitable for a wide range of acoustic environments. In addition, the model has been minimally customized for the speaker recognition task and can in fact provide a general framework for a variety of speech processing tasks. Our preliminary results do indeed show great robustness of a similar scheme for automatic speech recognition. It is therefore essential to emphasize that the performance obtained with the cortical features is *solely a property of the features themselves* and is achieved without any noise compensation techniques. Our ongoing efforts are aimed at achieving further improvements by applying the described multi-resolution cortical analysis on enhanced spectral profiles obtained using speech enhancement techniques, which involve estimation of noise characteristics in various forms [29].

## Figures and Tables

**Table 1:** List of conditions for NIST 2010 *extended core* task

Condition	Description
1	Microphone training, same microphone testing
2	Microphone training, different microphone testing
3	Microphone training, telephone testing
4	Microphone training, telephone conversation recorded with room microphone testing
5	NVE telephone training, NVE telephone testing
6	NVE telephone training, HVE telephone testing
7	Microphone training, HVE telephone testing
8	NVE telephone training, LVE telephone testing
9	Microphone training, LVE telephone testing

NVE, LVE, and HVE stand for normal, low, and high vocal effort, respectively.

**Table 2:** Average ASV performance (EER, %) as a function of noise type and condition

Cond.	Babble (0–24 dB)		White (0–24 dB)		Subway (0–24 dB)		Reverb (200–1,200 ms)	
			
	MFCC	Cortical	MFCC	Cortical	MFCC	Cortical	MFCC	Cortical
1	6.43	5.24	12.3	7.93	8.5	6.97	9.39	6.87
2	11.47	9.09	18.06	12.71	14.1	11.55	13.88	9.58
3	7.33	6.31	10.86	8.87	8.89	7.84	16.70	14.96
4	7.86	6.78	14.88	10.87	10.36	8.79	12.66	9.78
5	6.39	5.98	8.52	7.55	7.70	6.89	14.05	10.59
6	10.45	9.95	11.04	10.48	11.12	10.39	20.00	16.14
7	9.62	10.64	13.30	12.67	10.67	12.09	19.21	16.65
8	5.19	5.46	7.19	6.48	6.00	6.04	12.78	9.48
9	6.84	6.00	13.58	11.46	8.94	8.59	9.43	6.84

**Table 3:** Average ASV performance (Miss-10 metric, %) as a function of noise type and condition

Cond.	Babble (0–24 dB)		White (0–24 dB)		Subway (0–24 dB)		Reverb (200–1,200 ms)	
			
	MFCC	Cortical	MFCC	Cortical	MFCC	Cortical	MFCC	Cortical
1	5.3	3.2	17.2	7.9	9.4	6.0	9.1	4.6
2	16.6	10.8	31.3	18.1	22.9	16.4	19.8	9.5
3	6.2	4.4	14.0	9.0	9.2	6.9	26.5	21.9
4	8.1	5.7	22.9	13.7	13.0	9.9	16.9	9.9
5	4.9	3.7	9.4	6.7	7.0	5.3	20.3	11.8
6	11.6	10.2	13.6	11.4	13.2	11.2	35.6	25.1
7	11.8	12.5	18.6	16.6	13.0	16.5	34.2	26.6
8	3.2	2.9	7.7	5.0	4.7	3.9	17.0	9.4
9	6.5	5.5	21.4	15.4	11.0	9.9	9.0	4.6

**Table 4:** Average ASV performance (quadratic DCF metric) as a function of noise type and condition

Cond.	Babble (0–24 dB)		White (0–24 dB)		Subway (0–24 dB)		Reverb (200–1,200 ms)	
			
	MFCC	Cortical	MFCC	Cortical	MFCC	Cortical	MFCC	Cortical
1	0.129	0.098	0.371	0.211	0.208	0.147	0.219	0.150
2	0.247	0.183	0.506	0.342	0.356	0.266	0.353	0.221
3	0.149	0.116	0.282	0.196	0.199	0.155	0.481	0.403
4	0.176	0.136	0.446	0.287	0.276	0.199	0.317	0.237
5	0.139	0.114	0.186	0.150	0.171	0.136	0.379	0.262
6	0.236	0.230	0.255	0.240	0.245	0.241	0.557	0.457
7	0.231	0.240	0.452	0.362	0.302	0.300	0.543	0.486
8	0.105	0.088	0.154	0.119	0.122	0.105	0.331	0.212
9	0.134	0.105	0.405	0.288	0.218	0.175	0.220	0.145

## Footnotes

a We constraint the range of spectral modulations to 4 CPO, which covers more than 90% of the entire spectral modulation energy in speech and is most important for speech comprehension [7].

b The difference in MI levels between the speech message and speaker identity may be attributed to the observation that the speech signal encodes more information about the underlying linguistic message than about the speaker.

c In addition to the MI analysis, we performed an empirical test regarding use of 0.25 CPO filter. An experiment was run on clean data with Ω_*c*_ = {0.25, 0.5, 1.0, 2.0} CPO and yielded a 3.4% EER—a decrease of performance compared with 2.7% EER for the system that used Ω_*c*_ = {0.5, 1.0, 2.0, 4.0} CPO.
